# Fauna and Ecology of Macromycetes (Basidiomycota) in the Arctic Tree and Shrub Ecosystems of Central Siberia

**DOI:** 10.3390/jof10060435

**Published:** 2024-06-19

**Authors:** Sergey Sergeevich Kulakov, Andrey Ivanovich Tatarintsev, Denis Aleksandrovich Demidko, Natalia Pavlovna Khizhniak

**Affiliations:** 1 Institute of Forest Siberian Branch of the Russian Academy of Sciences, Reshetnev Siberian State University of Science and Technology, 660037 Krasnoyarsk, Russia; tatarintsevai@sibsau.ru (A.I.T.); sawer_beetle@mail.ru (D.A.D.); natalia-mx@mail.ru (N.P.K.); 2 Division of the Federal Research Center, Krasnoyarsk Science Center of the Siberian Branch of the Russian Academy of Sciences, Sukachev Institute of Forest Siberian Branch of the Russian Academy of Sciences, Akademgorodok 50/28, 660036 Krasnoyarsk, Russia

**Keywords:** Krasnoyarsk Krai, Norilsk, larch forests, macromycetes, species composition, trophic structure

## Abstract

**Simple Summary:**

The Arctic tree and shrub ecosystems of Central Siberia are distinctive in their ability to perform crucial biosphere functions. However, such forests have been the subject of relatively limited research. The understanding of the dynamics of these forests, including their composition and structure, is of significant relevance for the conservation of permafrost ecosystem biodiversity. Nevertheless, the species composition of the mycobiota of this region remain under investigation. The aim of the present study is to determine the macromycetes diversity in the main ecotopes of the Krasnoyarsk Arctic (Norilsk). An understanding of the ecological attributes of macromycetes within these ecosystems is essential for understanding of the decomposition of organic matter, the absorption of nutrients by trees and shrubs, and other fundamental ecological processes. Moreover, basidiomycetes may be utilized as indicator species in ecological studies.

**Abstract:**

The research was aimed at studying the taxonomic diversity, habitat specialization, and trophic characteristics of mycobiota, including Basidiomycota, in the northern ecosystems of the Krasnoyarsk Krai (Central Siberia) near Norilsk. Larch forests and woodlands in the Siberian permafrost zone are distinctive and Basidiomycota, as a component of these ecosystems, plays an essential role in their functioning. Currently, there is a paucity of information about this group in Arctic ecosystems, both in terms of floristic and ecological aspects. Seventy species of macromycetes belonging to different trophic groups were discovered and identified. Only 15% of species occur regularly, while most species are found rarely or only once. The identified species belong to 44 genera, 25 families, and 8 orders, which are included in the class Agaricomycetes. The leading families in terms of the number of species are Russulaceae, Polyporaceae, Tricholomataceae, Suillaceae, Strophariaceae, and Cortinariaceae. Mycorrhizal fungi and wood decay fungi dominate the structure of mycobiota of the study area (the total share is 71%). The rest of the species (29%) are fungal decomposers inhabiting plant litter, the forest floor, and humus. The largest number of species occur in forest ecosystems, which are dominated by mycorrhizal and wood decay fungi (up to 70%), which are trophically associated with woody plants and debris. The fungal decomposers inhabiting plant litter, the forest floor, and humus dominate (about 80%) in the species composition of tundra, where, in the absence of woody substrate, wood decay fungi have not been found at all. The species richness of tree and shrub Arctic ecosystems is low, yet the taxonomical and ecological structure of Basidiomycota is similar to that observed in taiga and temperate forests. These data permit a more comprehensive description of the biodiversity of the Arctic and may prove useful in studying biological processes in these ecosystems.

## 1. Introduction

Larch forests and woodlands (low-density forests where trees and shrubs form a light canopy) in the Siberian permafrost zone are unique. They perform crucial biosphere functions and are a source of various natural resources. Despite their wide geographical distribution, such forests have been studied unevenly. This is especially true for sub-tundra (boundary region between tundra and boreal forests) and northern taiga forest ecosystems. Notably, their ecological role is much more significant than their resource potential [1,2,3]. Understanding high-latitude Siberian forest dynamics, as well as their composition and structure, is of high relevance. For the conservation of permafrost ecosystem biodiversity, a scientific approach to their sustainable management and resource protection is needed [4]. Silvicultural features of such forests have been studied by many Russian researchers [5,6]. However, the species composition and structure of mycobiota of this region are still under research [7,8,9,10,11,12,13]. An understanding of the ecological attributes of macromycetes within these ecosystems is essential for gaining insight into their underlying processes, such as the decomposition of organic matter, the absorption of nutrients by trees and shrubs, and so forth. 

The available literature does not provide complete information on the mycobiota of Siberia, especially its northern part. The most studied is the Altai-Sayan mountainous ecoregion [14,15,16,17,18,19,20,21,22]. Karatygin and colleagues provided an annotated list of fungi in “Russian Arctic Fungi” [7]. The book combines the research of multiple authors who studied the Russian North, including Central Siberia (Krasnoyarsk Krai). However, the book in question contains only fragmentary information about basidiomycetes for the region under study. The species composition of this taxonomic group is incomplete, and there is a paucity of information about their ecology. Notably, one of the study areas, described in “Russian Arctic Fungi”, is located 230 km north of Norilsk. Poor knowledge of the mycobiota of the Russian North makes it difficult to reveal patterns in the geographical distribution of fungi. The available data on the ecology of agaric fungi mostly refers to their caprophores, which have a narrower ecological amplitude than mycelium [23]. Furthermore, basidiomycetes may serve as indicator species in ecological studies. The presence of their fruiting bodies, which can be readily and rapidly identified, indicates the occurrence of specific processes within the plant community. Ascomycetes, which are represented in the Arctic tree and shrub communities by numerous species, are a less optimal choice due to the brief lifespan of their fruiting bodies. Another reason they are an inferior indicator species is that they are challenging to rapidly identify in the field. For this reason, we excluded Ascomycota from further consideration. 

The aim of the present study is to determine the macromycetes diversity in the main ecotopes of the Krasnoyarsk Arctic (Norilsk). The subject of our investigation is the ecosystem of trees and shrubs. In this study, we concentrated on basidiomycetes, a group of fungi that includes numerous species closely related to trees and shrubs.

## 2. Material and Methods

### 2.1. Study Area

The study area belongs to the Norilsk-Evenki Ecoregion [24], covering an area between Nizhnyaya Tunguska in the south and Khatanga in the north. The most elevated part of the Central Siberian Plateau (table mountains and a plateau in the center) is also located in the study area. The vegetation is represented by mountain larch forests (forest floor dominated by reindeer moss and shrubs), woodlands, shrub communities at the tree line (alder and birch), and mountain tundra. There is no vegetation at the highest elevations. 

The study area is located in the subarctic (boreal) climate zone. An Arctic air mass determines the climate of the region. The study area is dominated by sparse forests and tundra biomes due to continuous permafrost [25]. The number and nature of seasons are based on the polar day and polar night. In Norilsk, for example, polar day lasts 68 days, and polar night lasts 45 days. The warmest month is July (average monthly temperature is 14 °C) (Norilsk), and the coldest month is January (average monthly temperature is minus 25 °C) (Kayerkan). The amplitude of air temperature variations is about 87° [26,27]. The average annual precipitation ranges from 400 mm (Norilsk) to 700 mm (Talnakh). Rain/snow falls mainly in July–October; precipitation exceeds evaporation, generating wet soil conditions. The annual snow season typically lasts from October to May (240–260 days) [26]. Weather conditions during the growing season determine not only the abundance and diversity of agaricoid fungi but also their fruiting. Despite stressful environmental conditions, macromycetes do form fruit bodies [28,29,30,31,32,33].

Field studies were conducted at four 1 ha (100 × 100 m) research plots (RP), placed in the most typical biotopes for the study area. The research plots’ characteristics are given below. 

**Figure 1:**
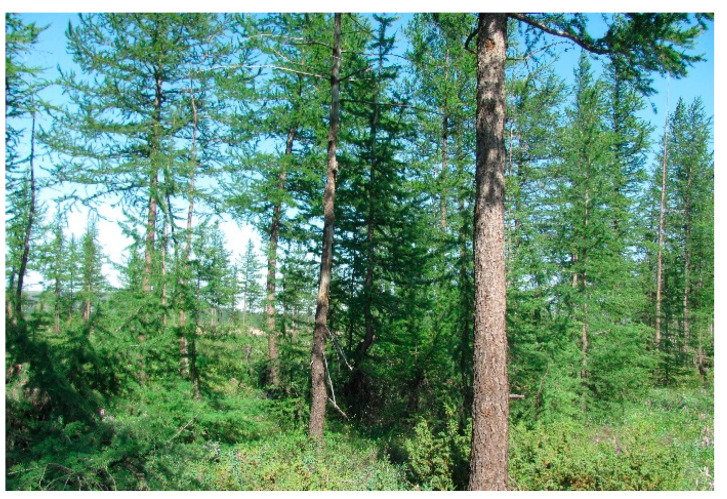
The general view of larch–alder forest (RP I).

Research plot I. Larch–alder forest (dominant tree species is *Larix sibirica*; dominant shrub species is *Duschekia fruticosa*). The field layer is dominated by horsetail ferns (mostly, *Equisetum pratense*) and herbs (*Anthoxanthum alpinum* prevail). The research plot is located 51 km west of Norilsk and 34 km west of Kayerkan: in the upper part of a southern convex–concave slope in the Bolgokhtokh River valley. The research plot I is located 50 m above sea level (Figure 1). 

**Figure 2:**
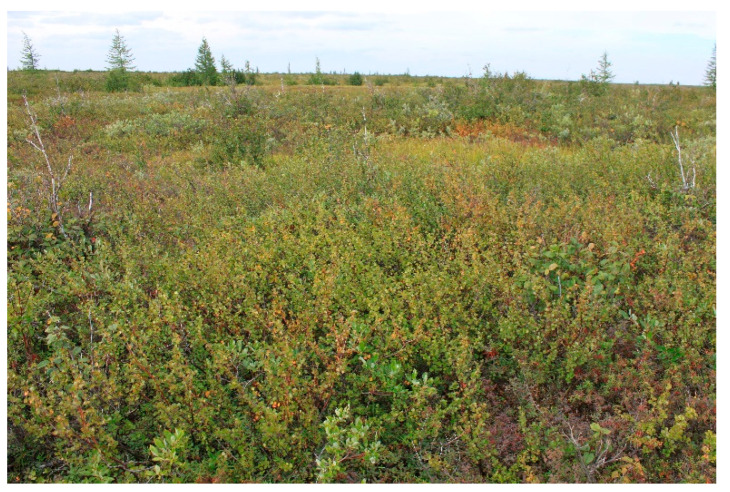
The general view of larch woodland (RP II).

Research plot II. Larch woodland. *Larix sibirica* mean high is 5–6 m (low canopy density). Shrubs cover about 70% of the research plot (dominant species is *Betula nana*). The research plot is located 51 km west of Norilsk and 33 km west of Kayerkan: on the terrace of the Bolgokhtokh River valley. The relief is mosaic: 60% of the research plot is micro hills (covered by subshrubs and lichens), and 40% of the research plot is micro depressions (covered by sedges and mosses). The research plot II is located 76 m above sea level (Figure 2).

**Figure 3:**
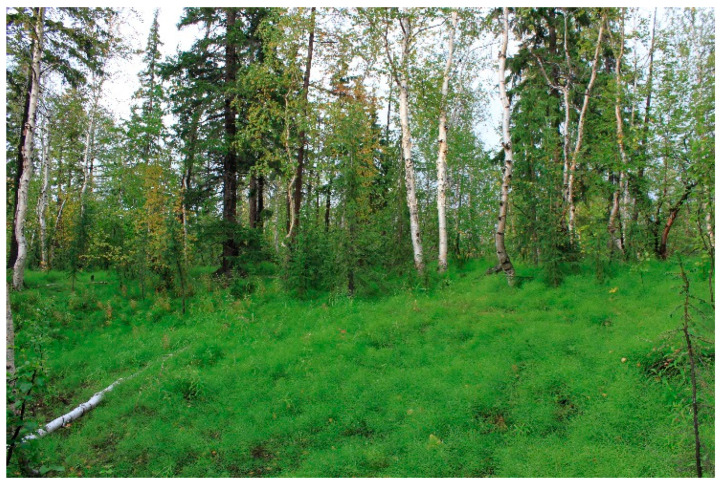
The general view of birch–spruce–larch forest (RP III).

Research plot III. Birch–spruce–larch forest. The field layer is dominated by horsetail ferns (mostly, *Equisetum pratense*). The research plot is located 1.5 km southeast of Talnakh and 15 km northeast of Norilsk: on the watershed of the Talnakh and Listvyanka rivers. There are both hills and lowlands on the research plot. Surface depressions are swampy or filled with thermokarst lakes. The research plot III is located 46 m above sea level (Figure 3).

**Figure 4:**
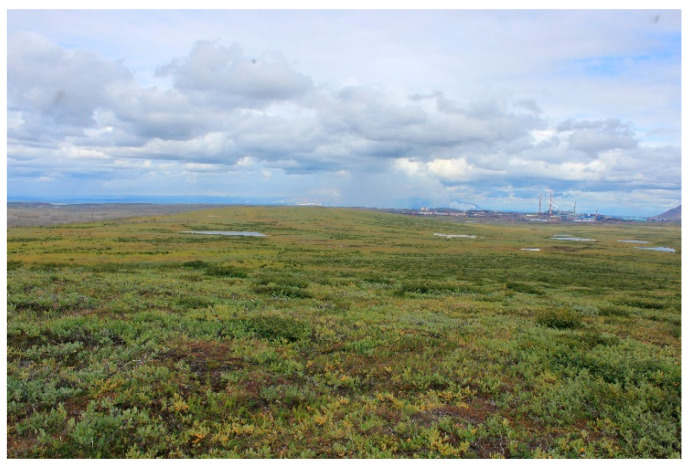
The general view of tundra (RP IV).

Research plot IV. Tundra covered by shrubs, subshrubs, and sedges. The research plot is located 6 km north of Talnakh and 23 km northeast of Norilsk: on the second above-floodplain terrace of the Kharaelakh River valley. The relief is represented by a gentle (3–4°) northwestern slope. The research plot IV is located 163 m above sea level (Figure 4).

Table 1 shows GPS coordinates for the research plots and brief species composition of studied plant communities.

### 2.2. Data Collection and Analysis

The material was collected in July–August 2018–2021 in the basin of the Bolgakhton and Norilsk rivers. The study was conducted using cruise method. Macromycetes were identified by their fruit bodies. The fruit bodies (basidiomas) were collected over a month at 10-day intervals. Common and easily identified fungi were recorded in a field diary. Specimens, that were difficult or impossible to identify in the field, were collected for the herbarium. Preparation of fungi specimens for deposit as herbarium followed standard methods [34,35,36]. Each fungi specimen was placed in a separate container and was assigned a unique number. For each herbarium specimen, we also recorded the following information: collection location, research plot number, habitat, frequency, and cover classes. A total of 104 herbarium specimens were collected. 

Identification of species was carried out in the Laboratory of Forest Cultures, Mycology, and Phytopathology of Sukachev Institute of Forest of Siberian Branch of the Russian Academy of Sciences. Microstructure was studied on dried material using a Mikmed 2 microscope and a standard set of reagents (KOH 5%, Melzer’s reagent for determining the amyloid reaction). The studied samples are stored in the herbarium of Sukachev Institute of Forest SB RAS.

The trophic level and habitat [12,16,17,18] of the identified macromycetes were determined according to Kovalenko classification, information about belonging to a trophic group is given according to [37]: 

I. **Decomposers:**

- On plant litter—Fd (folia desecta);
- On forest floor—St (stramentum);
- On humus—Hu (humus);
- On wood—Le (lignum epigaeum);
- On undamaged wood—Lei (lignum epigaeum integrum);
- On rotten wood—Lep (lignum epigaeum putridum);
- On roots and wood buried in soil—Lh (lignum hupogaeum);
- On mosses—M (muscu);
- On fungi fruit bodies—Mm (macromycetes);
- On excrements—E (excrementum).

II. **Simbionts:**

- Mycorrhizal fungi—Mr (mycorrhiza).

III. **Parasites:**

- Facultative parasites on trees and shrubs—P (parasitum).

To assess the distribution of macromycetes within ecotopes, generally accepted scales of frequency and cover were used [38]. The scales characterize the spatial arrangement of fruit bodies and provide a quantitative and qualitative assessment of the proportion that species take in a fungi community composition in various ecological and trophic groups. 

**Frequency classes:**

Very rare (rarissime): 1–2 sporocarps (localities).
Rare (raro): from 3 to 10 localities.
Rather frequent (saepe): more than 11 localities.
Very frequent (saepissime): more or less evenly over the entire area.

**Cover classes:**

Fruit bodies occur singly—1.
Fruit bodies occur in small groups. The number of fruit bodies in a group varies from 10 to 50—2.
Fruit bodies occur in large groups (from 50 to 100 or more) or distributed evenly over the entire area—3.

Fungal taxonomy and their scientific names are given in accordance with the system adopted in the 10th edition of the Ainsworth and Bisbee Dictionary of the Fung [39], as well as the Index Fungorum databases (http://www.indexfungorum.org accessed on 16 June 2024) [40] and Mycobank (http://www.mycobank.org accessed on 16 June 2024) [41].

## 3. Results

The present research resulted in identifying 70 species of macromycetes in the study area (Table 2). Most species (about 85%) were studied during the growing season of 2018 due to intensive basidioma formation.

Table 3 shows a list of fungi species, identified in the study area, indicating their trophic level, habitat, and frequency/cover classes.

**Figure 5:**
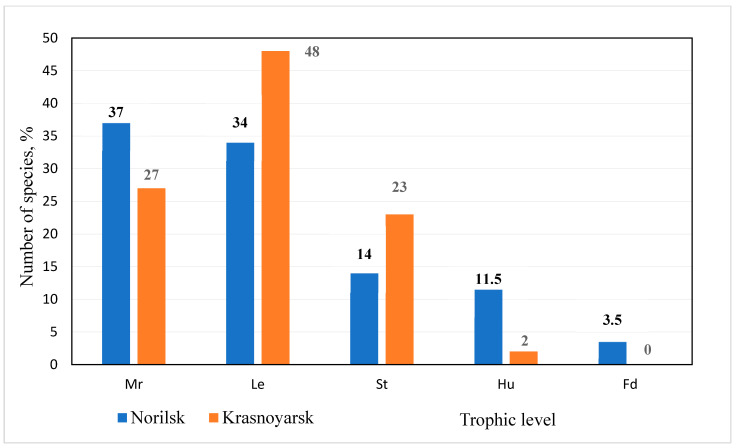
Trophic structure of macromycetes in the study area (near Norilsk) and near Krasnoyarsk (according to [42]): Mr—mycorrhizae; Le—decomposers on wood; St—decomposers on forest floor; Hu—decomposers on humus; Fd—decomposers on plant litter.

Figure 5 shows a relative distribution of macromycete species (identified near Norilsk) by trophic-level groups. We also suggest using data on the trophic structure of macromycetes in the southern taiga forests (near Krasnoyarsk) for comparative analysis [42].

Table 4 shows a quantitative assessment of the species composition and trophic structure of macromycete communities in the study area.

## 4. Discussion

The findings of the study permit the formulation of generalizations regarding the basidiomycete flora of the study area and their ecological role in the tree and shrub communities of the Arctic. 

The identified macromycetes (70 species) belong to 44 genera, 25 families, and 8 orders included in the subclass Agaricomycetidae (Table 2 and Table 3). The order Agaricales dominates by the number of species (34 species), followed by Russulales (14 species), Polyporales (9 species), and Boletales (8 species), making up a total of 93% of the species in the identified mycobiota. The remaining orders are represented by a few species of macromycetes (1–3 species). The six leading families, uniting 52% of the species of the studied biota, include Russulaceae (with the largest number of species—14), followed by Polyporaceae (6 species), Suillaceae, and Tricholomataceae (5 species each), Cortinariaceae and Strophariaceae (4 species each). The remaining families are represented by a smaller number of species, including 11 families represented by one species only. 

The species of fungi we discovered is not endemic to the Arctic [42]. Our research indicates that they can thrive in cold climates, including in the northern regions of Central Siberia. 

Macromycetes are trophically associated with vegetation: some directly interact with trees and shrubs, and some inhabit detritus, which is dominated by plant organic matter [43]. Thus, the species diversity of macromycetes largely depends on the vegetation diversity. Therefore, the largest number of fungi species were found within forest ecosystems (RP I and III), characterized by a richer species composition of plant communities (Table 4).

Mycorrhizal fungi and wood decay fungi dominate in the study area (Figure 5), which is also typical for the trophic structure of the macromycetes in the southern taiga [42] and temperate [23] forests. The relative number of species of these trophic levels in the northern latitudes (study area) is similar (37% and 34%, respectively), with a slight predominance of mycorrhizal fungi. In the southern taiga forests, wood decay fungi significantly exceed mycorrhizal fungi by the number of species [42]: 48% and 27%, respectively. The lower abundance of wood-associated fungal species compared to taiga forests (Figure 5) can be attributed to the smaller role of tree species in the communities under study and the simplified structure of the tree layer. Southern taiga forest communities, being of higher species diversity, provide a greater variety of wood habitat, compared to northern taiga forests and woodlands. Mycorrhizal and wood decay fungi play a crucial role in forest ecosystems. Mycorrhizal fungi form symbiotic relationships with the roots of trees, increasing their growth and productivity and contributing to an increased nutrient uptake (primarily carbon) by tree biomass. Wood decay fungi, on the other hand, contribute to carbon emissions by decomposing woody debris.

Mycorrhizal fungi occurred in all research plots (Table 4). In the larch woodland (RP II), they take 60% of the species composition of macromycetes. In typical northern taiga forest communities (RP I and III), mycorrhizal fungi take 39% and 31%, respectively. The most numerous among mycorrhizal fungi were species of the family Russulaceae (14 species), and the genus *Lactarius* (9 species) (Table 2 and Table 3). *L. vietus* (Fr.) Fr. is common, while *L. repraesentaneus* Britzelm. Pers. and *L. pubescens* Fr. occur less frequently. The number of species of the genus *Russula* is relatively small, among which there are species associated exclusively with birch and species with a wide range of symbiont partners. *R. paludosa* Britzelm is the most common species in all research plots. Most species are ecologically flexible and capable of living in various habitats since they can form mycorrhizae with a wide range of woody plants.

Wood decay fungi occur in those plant communities, where a tree layer is present (Table 3). Wood decay fungi take the largest proportion in the species composition of the mycobiota in the birch–spruce–larch forest (RP III), which is explained by increased habitat diversity relative to other forests. Special mention should go to *Armillaria mellea* s.l, which prefers a parasitic lifestyle but is also able to feed saprotrophically (facultative decomposers). There were also sterile conks of *Inonotus obliquus* (Fr.) Pilát found on some living trees. Specialist fungi, that are able to live in one habitat only, are rare. For example, the host range of *Fomitopsis betulina* (Bull.) and *I*. *obliquus* is restricted exclusively to birch species. *Fomitopsis pinicola* (Sw.) P. Karst, on the other hand, colonizes wood of many deciduous and coniferous species (sometimes on weakened living trees). Fruit bodies of this species, along with *Bjerkandera adusta* and *Fomes fomentarius*, are quite common in forest communities.

Fungal decomposers inhabiting plant litter, forest floor, and humus together include about 30% of the species in the macromycete biota of the study area (Figure 5), which is consistent with previously obtained data for the southern taiga [42] and temperate [23] forests. They act as an important link in the nutrient cycle and improve soil fertility by decomposing plant residues. Fungal decomposers often exhibit broad trophic specialization, so their belonging to ecological and trophic groups cannot always be determined accurately. The proportion of species belonging to studied trophic-level groups is unequal within the research plots (Table 3). The share of fungal decomposers inhabiting plant litter, forest floor, and humus is 25–30% in forest ecosystems and woodlands (RP I–III); while in tundra it reaches 78% (RP IV) with the dominance of humus saprobionts (67%). Tundra biotopes are dominated by macromycetes inhabiting forest floor and humus, due to the presence of plant litter and the absence of woody substrate necessary for wood-decaying fungi (so no wood-decaying fungi was found there). 

The advantages and disadvantages of metabarcoding (ITS sequencing) of fungi are described in [44,45]. One clear advantage is the ability to identify a larger number of species compared to classical methods for studying fungal communities. However, for high latitudes, the use of this approach is complicated by the lack of sufficiently complete reference databases. A more universal technical problem is the difficulty in recognizing taxa due to either too high or insufficient intraspecific variability in fungi [44]. 

Nevertheless, it seems reasonable to compare our results with data on fungal diversity obtained using DNA barcoding. It was unexpected that the taxonomic diversity of mycorrhiza-forming basidiomycetes established in a study of Arctic communities involving larch [46] was found to be lower than that obtained in our studies. This work revealed the presence of only nine operational taxonomic units (OTUs) in the communities under study, in contrast to the 37 species we identified (Figure 5). This discrepancy is likely due to the greater diversity of plant communities that were studied in this work compared to that of [46]. A total of 115 amplicon sequence variants (ASVs) belonging to the basidiomycetes were identified in the soils of Spitsbergen, representing 13.07% of the total fungal diversity [47]. This represents a notable increase compared to our findings, although to some extent it can be attributed to the more diverse conditions under which the soil samples were collected for analysis. 

A comprehensive examination of the North American Arctic, conducted in tundra communities, led to the identification of 1834 OTUs, of which 486 were classified as basidiomycetes, belonging to 36 families [48]. A comparison of our results with data for zone E, which is the most similar in climatic conditions to Norilsk, showed a high degree of similarity between them in the level of alpha diversity (73 OTUs versus 70 species in our research plots). A detailed analysis of the similarities in the structure of trophic groups is difficult due to the lack of this information in [48]. However, the data we know about the taxa found in the North American Arctic allows us to conclude that there are no sharp differences. It should also be noted that the distribution of the most typical fungal taxa found in [48] is not limited to the Arctic. This conclusion is entirely consistent with the data obtained. 

## 5. Conclusions

The diversity of macromycetes in the studied area is relatively limited at the species level but is comparable to that observed in more southern ecosystems at the genus-to-order level. The leading families in terms of the number of species are Russulaceae (14 species), Polyporaceae (6), Tricholomataceae (5), and Suillaceae (5). This represents the inaugural comprehensive data set on Basidiomycota in the Arctic zone of Central Siberia. The most common species are mycorrhizal fungi (37%), which form a symbiotic association with plant roots, and wood decay fungi (34%), which decompose woody debris. The rest (29%) are fungal decomposers inhabiting plant litter, the forest floor, and humus. 

The number of species and their habitat and trophic spectrum depend on the diversity of plant substrates. The largest number of species occur in forest biotopes dominated by mycorrhizal and wood decay fungi (up to 70%), that are trophically associated with woody plants and woody debris. The fungal decomposers inhabiting plant litter, the forest floor, and humus dominate (about 80%) in the species composition of tundra, where, in the absence of woody substrate, wood decay fungi have not been found at all. The ecological structure of the arctic fungal community is similar to that of the taiga and temperate forests. The data obtained can be utilized for the purpose of identifying the biological processes occurring in the Arctic ecosystems. 

The study of macromycetes in Northern Siberia revealed their species diversity and role in the unique high-latitude ecosystems. Since fungi form fruit bodies irregularly and their life cycle is short in such harsh conditions, it is impossible to identify all macromycete species, inhabiting northern ecosystems, at once [49]. Thus, further research on macromycetes in the study area is needed. 

## Figures and Tables

**Table 1:** Study locations and brief species composition of studied plant communities.

RP	Plant Community	Coordinates		Species Composition	
		Latitude	Longitude	Trees	Shrubs
I	Larch–alder forest	69°20′31.26″ C	86°51′57.06″ B	*Larix sibirica* (canopy density is 0.4–0.5)	*Duschekia fruticosa*, *Betula tortuosa* (cover 100% of the RP)
II	Larch woodland	69°20′36.58″ C	86°53′27.50″ B	*Larix sibirica* (canopy density is less than 0.1)	*Betula nana*, *Duschekia fruticosa* (cover 70% of the RP)
III	Birch–spruce–larch forest	69°28′20.20″ C	88°25′46.20″ B	*Larix sibirica*, *Picea obovat*, and *Betula tortuosa* in the II layer (canopy density is 0.4–0.5)	*Duschekia fruticosa* and *Juniperus sibirica* (grow in groups)
IV	Tundra	69°32′58.82″ C	88°28′2.03″ B	No trees	*Betula nana*, *Salix lanata*, *Salix pulchra* and *Duschekia fruticosa* (shrub layer is almost absent)

**Table 2:** Taxonomic composition of basidiomycetes (family and genus level) in the study area and number of species for each family on the research plots.

Family (Number of Genera/Number of Species)	Genus (Total Number of Species)	Including Species for Each RP			
		I	II	III	IV
Agaricaceae (2/3)	*Cystoderma* (2), *Lycoperdon* (1)	3		1	
Amanitaceae (1/1)	*Amanita* (1)				1
Bolbitiaceae (1/1)	*Pholiotina* (1)	1		1	
Cortinariaceae (1/4)	*Cortinarius* (4)	3	1	2	3
Entolomataceae (1/1)	*Entoloma* (1)			1	
Hydnangiaceae (1/1)	*Laccaria* (1)	1			
Hygrophoraceae (1/1)	*Hygrophorus* (1)	1	1	1	
Hymenogastraceae (3/3)	*Hypholoma* (1), *Galerina* (1), *Gymnopilus* (1)	2	1	1	
Inocybaceae (1/1)	*Crepidotus* (1)	1		1	
Marasmiaceae (1/1)	*Marasmius* (1)	1		1	
Mycenaceae (1/2)	*Mycena* (2)	1		1	1
Omphalotaceae (1/1)	*Gymnopus* (1)			1	
Physalacriaceae (2/2)	*Armillaria* (1), *Coprinopsis* (1)	1		1	
Pleurotaceae (1/1)	*Pleurotus* (1)	1		1	
Psathyrellaceae (2/2)	*Parasola* (1), *Psathyrella* (1)	1		1	
Strophariaceae (2/4)	*Kuehneromyces* (1), *Pholiota* (3)	2		2	1
Tricholomataceae (4/5)	*Clitocybe* (2), *Leucocortinarius* (1), *Paralepista* (1), *Tricholoma* (1)	2	1	2	1
Boletaceae (1/3)	*Leccinum* (3)	2	1	2	
Suillaceae (2/5)	*Boletinus* (1), *Suillus* (4)	4	2	3	1
Gloeophyllaceae (1/1)	*Gloeophyllum* (1)			1	
Hymenochaetaceae (3/3)	*Phellinus* (1), *Inonotus* (1), *Xanthoporia* (1)	2		1	
Fomitopsidaceae (2/3)	*Fomitopsis* (2), *Meripilus* (1)	1		2	
Polyporaceae (6/6)	*Bjecandera* (1), *Fomes* (1), *Lentinus* (1), *Neolentinus* (1), *Trametes* (1), *Trichaptum* (1)	3		4	
Russulaceae (2/14)	*Lactarius* (9), *Russula* (5)	6	3	5	1
Bankeraceae (1/1)	*Thelephora* (1)			1	
25	44/70	49	10	36	9

**Table 3:** Fungi species list and their ecological features.

Genus, Species	Trophic Level	Frequency	Cover	Habitat
1	2	3	4	5
Subdivision		BASIDIOMYCOTA		
Class		Agaricomycotina		
Subclass		Agaricomycetes		
Order		Agaricomycetidae		
Family		Agaricales		
Subdivision		Agaricaceae		
*Cystoderma amiantinum* (Scop.) Fayod.	St	Saepe	1	Mixed forest floor
*Cystoderma grannulosum* (Batsch: Fr.) Fay	St	Saepe	1	Ground, among deciduous and coniferous litter
*Lycoperdon pyriforme* Schaeff.	St	Rarissime	2	Ground, among deciduous and coniferous litter
Family Amanitaceae				
*Amanita regalis* (Fr.) Michae	Hu	Rarissime	1	Ground
Family Bolbitiaceae				
*Pholiotina dasypus* (Romagn.) P.-A. Moreau	Hu	Rarissime	1	Humus
Family Cortinariaceae				
*Cortinarius armillatus* (Fr.) Fr.	Mr	Rarissime	1	Moist places, at the edge of bogs, on hummocks, moss bedding
*Cortinarius evernius* (Fr.) Fr.	Fd	Rarissime	1	Moist places, near swamps, moss bedding
*Cortinarius mucosus* (Bull.) J. Kickx f.	Hu	Rarissime	1	Mixed dark coniferous forests, sphagnum bogs
*Cortinarius uliginosus* Berk.	Hu	Rarissime	1	Moist, swampy or seasonally flooded soils
Family Entolomataceae				
*Entoloma cetratum* (Fr.) M.M. Moser	Fd	Rarissime	1	Decaying litter
Family Hydnangiaceae				
*Laccaria laccata* (Scop.) Cooke	Hu, Lep	Saepe	1	Ground in forests or grasslands
Family Hygrophoraceae				
*Hygrophorus lucorum* Kalchbr	Mr	Raro	1	Ground near larch
Family Hymenogastraceae				
*Hypholoma capnoides* (Fr.) P. Kumm.	Le, Lh	Raro	2	Wood at different decay classes
*Galerina hypnorum* (Schrank) Kühner	St, Lep	Raro	1	Moss, well-rotten wood
*Gymnopilus liquiritiae* (Pers.) P. Karst.	Lep	Raro	1	Wood at different decay classes
Family Inocybaceae				
*Crepidotus mollis* (Schff.: Fr.) P. Kumm.	Le, Lep	Raro	1	Wood at different decay classes
Family Marasmiaceae				
*Marasmius wettsteinii* Sacc. and P. Syd.	St,	Raro	1	Coniferous litter
Family Mycenaceae				
*Mycena haematopus* (Pers.) P. Kumm.	Lep, Lh	Raro	2	Mossy stumps, lying deadwood
*Mycena flavoalba* (Fr.) Quél.	Hu	Raro	1	Ground in deciduous and coniferous forests
Family Omphalotaceae				
*Gymnopus confluens* (Pers.) Antonín, Halling and Noordel.	St, Lep	Raro	2	Litter, rotten stumps
Family Physalacriaceae				
*Armillaria mellea* (Vahl) P. Kumm.	Le, Lep	Raro	2	Wood at different decay classes
*Coprinopsis atramentaria* (Bull.) Redhead	St, Lep	Raro	2	Grass, stumps of deciduous trees
Family Pleurotaceae				
*Pleurotus ostreatus* (Jacq.) P. Kumm.	Lep	Raro	1	Wood
Family Psathyrellaceae				
*Parasola plicatilis* (Curtis) Redhead	Hu	Raro	1	Grasslands, along roads
*Psathyrella piluliformis* (Bull.) P.D. Orton	Lep	Rarissime	2	Woody debris
Family Strophariaceae				
*Kuehneromyces mutabilis* (Schaeff.) Singer and A.H. Sm.	Lep	Raro	2	Stumps of deciduous (less often—coniferous) trees
*Pholiota lenta* (Pers.) Singer	Lep	Raro	1	Well-rotten wood
*Pholiota squarrosa* (Vahl) P. Kumm.	Lep	Raro	1	Well-rotten wood
*Pholiota squarrosoides* (Peck) Sacc.	Lep	Raro	1	Well-rotten wood
Family Tricholomataceae				
*Clitocybe brumalis* (Fr.) Quél.	St	Raro	1	Litter, among feather mosses
*Clitocybe gibba* (Pers.) P. Kumm.	St	Raro	1	Litter, among feather mosses
*Leucocortinarius bulbiger* (Alb. and Schwein.) Singer	Mr	Saepe	1	Litter
*Paralepista gilva* (Pers.) Raithelh.	St	Raro	1	Litter, among feather mosses
*Tricholoma argyraceum* (Bull.) Gillet	Mr	Raro	1	Litter
Order Boletales				
Family Boletaceae				
*Leccinum aurantiacum* (Bull.) Gray	Mr	Raro	1	Mixed forests
*Leccinum scabrum* (Bull.) Gray	Mr	Raro	1	Birch-dominated and mixed forests
*Leccinum variicolor* Watling	Mr	Raro	1	Birch-dominated and mixed forests
Family Suillaceae				
*Boletinus spectabilis* (Peck) Murrill	Hu	Rarissime	1	Swampy soil in forests where larch grow
*Suillus granulatus* (L.) Roussel	Mr	Raro	1	Mossy and lichenous vegetation
*Suillus grevillei* (Klotzsch) Singer	Mr	Raro	1	Ground in forests where larch grow
*Suillus luteus* (L.) Roussel	Mr	Raro	1	Mossy vegetation
*Suillus viscidus* (L.) Roussel	Mr	Raro	1	In forests where larch grow
Order Gloeophyllales				
Family Gloeophyllaceae				
*Gloeophyllum sepiarium* (Wulfen) P. Karst.	Le, Lep	Raro	2	Stumps, standing and lying deadwood of coniferous trees
Order Hymenochaetales				
Family Hymenochaetaceae				
*Phellinus tremulae* (Bondartsev) Bondartsev and P.N. Borisov	Le, Lep	Rarissime	1	Living and dead aspen trunks
*Inonotus obliquus* (Fr.) Pilát (Чaгa)	Le	Raro	1	Living birch trunks
*Xanthoporia radiata* (Sowerby) Ţura, Zmitr.	Le, Lep	Raro	1	Weakened and dead deciduous trees
Order Polyporales				
Family Fomitopsidaceae				
*Fomitopsis betulina* Bull.	Le, Lep	Saepe	1	Living and dead birch
*Fomitopsis pinicola* (Sw.) P. Karst.	Le, Lep	Saepissime	1	Stumps and lying dead trees; occasionally act as parasitic fungi that feed on weakened trees
*Meripilus giganteus* (Pers.) P. Karst.	Le, Lei	Raro	1	Roots of deciduous trees
Family Polyporaceae				
*Bjerkandera adusta* (Willd.: Fr.) P. Karst.	Lep	Saepissime	2	Rotten wood
*Fomes fomentarius* (L.) J. J. Kickx	Le, Lep	Saepissime	1	Birch stumps and lying dead trees; occasionally act as parasitic fungi that feed on weakened trees
*Lentinus brumalis* (Pers.) Zmitr.	Le, Lep	Raro	1	Stumps and lying branches of various deciduous species
*Neolentinus lepideus* (Fr.) Redhead and Ginns	Le, Lep	Raro	1	Lying deadwood and stumps of coniferous trees; timber damaged by wood borers
*Trametes hirsuta* (Wulfen) Lloyd	Lep	Raro	1	Standing and lying dead trees and stumps
*Hirschioporus fuscoviolaceus* (Ehrenb.) Donk	Lep	Raro	1	Standing and lying dead trees and stumps of coniferous species
Order Russulales				
Family Russulaceae				
*Lactarius helvus* (Fr.) Fr.	Mr	Rarissime	1	Ground, among old litter
*Lactarius flexuosus* (Pers.) Gray	Mr	Rarissime	1	Ground, among old litter
*Lactarius torminosus* (Schaeff.) Gray	Mr	Raro	1	Ground in deciduous, coniferous and mixed forests
*Lactarius porninsis* Rolland	Mr	Rarissime	1	Ground, among old litter
*Lactarius pubescens* Fr.	Mr	Raro	1	Ground, among mossy litter
*Lactarius repraesentaneus* Britzelm.	Mr	Rarissime	1	Ground, among old litter
*Lactarius scrobiculatus* (Scop.) Fr.	Mr	Rarissime	1	Ground, among old litter
*Lactarius vellereus* (Fr.) Fr.	Mr	Saepe	1	Ground, among old litter
*Lactarius vietus* (Fr.) Fr.	Mr	Rarissime	1	Ground, among old litter
*Russula aeruginea* Lindblad ex Fr.	Mr	Rarissime	1	Ground, among old litter
*Russula claroflava* Grove	Mr	Rarissime	1	Ground, among old litter
*Russula exalbicans* (Pers.) Melzer and Zvára	Mr	Rarissime	1	Ground, among old litter
*Russula paludosa* Britzelm.	Mr	Raro	1	Ground, among mossy litter
*Russula xerampelina* (Schaeff.) Fr.	Mr	Rarissime	1	Ground, among old litter
Order Thelephorales				
Family Bankeraceae				
*Thelephora caryophyllea* (Schaeff.) Pers.	Mr	Rarissime	1	Ground in coniferous and mixed forests

**Table 4:** Species composition and trophic structure of macromycetes in the study area.

Plant Community	Number of Species	Distribution of Macromycetes by Trophic-Level Groups, %				
		Mycorrhizal Fungi (Mr)	Decomposers			
			On Wood (Le)	On Forest Floor (St)	On Humus (Hu)	On Plant Litter (Fd)
Larch–alder forest (RP I)	49	38.8	30.6	20.4	8.2	2.0
Larch woodland (RP II)	10	60.0	10.0	10.0	20.0	0
Birch–spruce–larch forest (RP III)	36	30.6	44.4	11.1	11.1	2.8
Tundra (RP IV)	9	22.2	0	11.1	66.7	0

## Data Availability

The original contributions presented in the study are included in the article, further inquiries can be directed to the corresponding author.
